# “*I knew what was going to happen if I did nothing and so I was going to do something*”: Faith, hope, and trust in the decisions of Canadians with multiple sclerosis to seek unproven interventions abroad

**DOI:** 10.1186/1472-6963-14-445

**Published:** 2014-09-30

**Authors:** Jeremy Snyder, Krystyna Adams, Valorie A Crooks, David Whitehurst, Jennifer Vallee

**Affiliations:** Faculty of Health Sciences, Simon Fraser University, 8888 University Dr, Burnaby, BC Canada; Department of Geography, Simon Fraser University, 8888 University Dr, Burnaby, BC Canada

**Keywords:** Medical tourism, Multiple sclerosis, CCSVI, Canada

## Abstract

**Background:**

Chronic cerebrospinal venous insufficiency (CCSVI) treatment is an unproven intervention aimed at relieving some of the symptoms of multiple sclerosis (MS). Despite limited evidence of the efficacy and safety of this intervention, Canadians diagnosed with MS have been traveling abroad to access this procedure as it is not available domestically outside of limited clinical trials. This paper discusses the experiences of Canadians with MS seeking CCSVI treatment abroad.

**Methods:**

This paper presents a secondary analysis of 15 interviews with participants who had gone abroad for CCSVI treatment. Interviews were conducted over the phone between October 2012 and December 2012. All interviews were digitally recorded and transcribed verbatim. Transcripts were hand coded for: 1) why CCSVI treatment was sought and where it was obtained; 2) the role of having hope for a cure in seeking CCSVI; 3) the impact of MS on everyday life; and 4) the role other people played in the decision to go abroad.

**Results:**

The authors identified loss of faith, hope, and trust as themes emerging from the transcripts. The participants experienced a loss of faith with the Canadian health system and especially the neurologists who were responsible for their care and the classification of MS as a neurological disease. Access to CCSVI treatment abroad generated hope in these participants, but they were cautious in their expectations, focusing on symptom management rather than a cure. Trust in their caregivers abroad was generated through the recommendations of other MS sufferers and the credentials of their caregivers abroad.

**Conclusions:**

By deciding to seek an unproven intervention abroad, these individuals took on responsibility for their care from the Canadian health system. While evidence of the efficacy of CCSVI treatment is limited, the participants felt that they were making a rational care decision, focusing on the empowerment and renewed hope generated by seeking this intervention. Health professionals and policy makers globally should consider the causes of loss of faith in their domestic care systems and balance the benefits of empowerment and renewed hope against concerns that unproven interventions may create new health risks.

## Background

Canadians have long traveled abroad for medical care as medical tourists [1]. While many of these individuals travel abroad to avoid wait times for necessary care or to obtain lower prices for elective procedures, others seek procedures not available domestically because of a lack of capacity or because they have not been approved for domestic use. A wide range of unproven medical interventions and equipment draw Canadians abroad for care, including stem cell interventions and new forms of hip replacements [1, 2]. Recently, some Canadians’ interest in obtaining care abroad has been spurred by an unproven intervention for multiple sclerosis (MS) known as chronic cerebrospinal venous insufficiency (CCSVI) treatment. Following a prolonged debate as to whether the Canadian government would fund access to this intervention, a negative decision was reached, effectively blocking domestic access to CCSVI. As a result, some Canadians with MS have decided to travel abroad in order to access this care, thus circumventing restrictions placed by their domestic government [3].

MS is a disorder of the central nervous system with undetermined causes. It leads to damage to the central nervous system through an autoimmune response, resulting in a variety of symptoms including vertigo and changes in vision, sensory sensation, energy levels, and motor function [4]. In 2009, a new theory of the pathology of MS was presented by Paolo Zamboni who suggested abnormal cranial venous outflow or CCSVI may be present in all people who have MS [5]. According to Zamboni’s theory, this abnormal outflow is responsible for at least some of the symptoms associated with MS [5]. Treatment for this proposed condition is available, involving expanding the blocked or narrowed vein (typically the jugular) using venoplasty or stents [6].

Since 2009, researchers have investigated the suggested association between CCSVI and MS. There have been no subsequent reported results with the high correlation of 100% seen in the initial Zamboni study. A cross-sectional study by Zivadinov et al. [7] found that 56% of MS participants qualified for a CCSVI diagnosis while prevalence in healthy persons was 22% and persons with other neurological disorders had 42% prevalence with CCSVI. However, no randomized control trials have provided evidence of the efficacy of CCSVI treatment for the relief of the symptoms of MS [8–13]. Most recently, studies of 199 and 177 individuals with MS respectively failed to find a connection between CCSVI and MS [9, 14].

Canada has one of the highest incidences of MS in the world, with over 95,000 cases diagnosed in 2013 [15, 16]. Despite the lack of evidence linking CCSVI treatment with symptom relief in individuals with MS, there has been considerable pressure within Canada to make this intervention available to interested individuals [17, 18]. Canada’s public health care system, which is administered separately by each province/territory as per the national Canada Health Act, provides medically necessary care to citizens through interventions determined to be efficacious and cost effective [19]. Treatment of CCSVI for Canadians with MS has not been approved in the public system due to the lack of clear evidence of efficacy, though some Canadian provinces and the federal government have funded clinical trial participation for their residents both in Canada and abroad [20]. The Canada Health Act heavily restricts the provision of private medical care in the country, meaning that CCSVI treatment cannot be offered domestically for Canadians wishing to pay privately for such care. While federal and provincial funding for clinical trials on CCSVI has been approved, these trials currently provide very limited opportunities for Canadians to receive CCSVI treatment domestically [18]. As a result, Canadians with MS wishing to access CCSVI treatment are left with the options of seeking to become one of the few individuals allowed to enroll in clinical trials on CCSVI treatment or privately seeking interventions outside of the country as medical tourists.

Those Canadians who choose to travel abroad to obtain CCSVI treatment must arrange for this intervention on their own and pay for it out-of-pocket [21, 22]. In some cases, individuals have reported symptom relief following CCSVI treatment [23]. In the province of British Columbia, for example, half of those persons participating in a registry for CCSVI treatment recipients reported symptom relief [24]. This symptom relief is often not permanent, however, requiring individuals to travel abroad repeatedly for CCSVI treatment [24]. Other Canadians who have already gone abroad for treatment have faced complications and difficulties obtaining follow up care for their MS from their regular health care providers [25]. In extreme cases, obtaining CCSVI treatment abroad has led to the death of Canadians. For example, one Canadian man died following CCSVI treatment received in Costa Rica after experiencing difficulty obtaining treatment for complications following the procedure upon return home to Canada [26]. A second Canadian patient who received CCSVI treatment in the United States died from complications from the procedure as well [26]. These setbacks have led to charges that the Canadian media engaged in ‘hope mongering’ that has encouraged people with MS to engage in a risky and unproved therapy [27]. Nonetheless, MS advocacy groups have become highly organized through social media and have been successful in influencing the Canadian political debate on the funding of CCSVI treatment in Canada [3]. Despite the lack of clear evidence connecting MS and CCSVI, Canadians continue to advocate for domestic access to CCSVI treatment and to travel abroad for CCSVI treatment in the hope that it will at least temporarily relieve some of the symptoms of their disease.

In this paper, we present findings from 15 semi-structured interviews with Canadians with MS who sought CCSVI treatment abroad. We specifically examine issues central to their decisions to go abroad for this intervention. This paper serves to shed light on the experiences of Canadians who have received CCSVI treatment as medical tourists and will serve to augment research on the global health services practice of medical tourism.

## Methods

This analysis is part of a larger study aiming to develop an informational tool for better informing Canadians about ethical concerns related to medical tourism [28]. As part of this study interviews were conducted with Canadians who had previously gone abroad as medical tourists to examine their decision-making processes and the usefulness of such a tool. During the initial analysis of these interviews, distinct perspectives emerged on the decision-making process from medical tourists diagnosed with MS and traveling abroad for CCSVI (n = 15) compared to those traveling for other procedures (n = 9) (e.g., gastrectomy, cholecystectomy, lumbar disc replacement). This paper presents a secondary analysis that examines the cases of only those 15 interviewees who had gone abroad for CCSVI treatment. 9 participants identified as female and 6 as male. Their age ranged from 28 to 65 with a median age of 50. Participant income included less than CDN$30,000 (n = 3); $30,000-50,000 (n = 4); $50,000-80,000 (n = 4); and more than $80,000 (n = 3) with one participant not responding. They had completed high school (n = 1), taken some college courses (n = 7), completed a secondary degree (n = 5), or taken some graduate courses (n = 2). These individuals lived in 5 different Canadian provinces and went abroad to Bulgaria, Egypt, India, Mexico, Poland, and the United States between 2010 and 2012 for CCSVI treatment.

### Recruitment

Following receipt of ethical approval from the Office of Research Ethics at Simon Fraser University, we recruited former Canadian medical tourists to participate in these interviews using: 1) advertisement on Craigslist; 2) advertisement in a Vancouver-based newspaper; 3) posting invitations to participate in online medical tourism fora; and 4) snowball sampling through interview participant networks and past research participant networks. Interested individuals were asked to contact either a toll-free number or email address for more information about the study and assessment of eligibility. Eligibility was assessed according to four inclusion criteria: 1) 18 years or older at the time of going abroad for medical care; 2) a holder of a provincial/territorial medical card; 3) someone who had accessed a surgical procedure outside of Canada that was neither a transplantation nor a reproductive surgery (as these procedures often involve third parties and raise distinct ethical issues); and 4) someone who paid privately for the medical procedure sought abroad. Those deemed eligible for participation were provided with a consent form to read and sign before beginning the interview. Verbal confirmation of informed consent was also obtained at the beginning of the interview and participants were assured of their anonymity.

### Data collection

Interviews were conducted between October 2012 and December 2012. On-going recruitment occurred during these months with the intention of completing as many interviews as possible before ceasing recruitment at the end of this time period. Interviews lasted on average 45 minutes, with the shortest lasting approximately thirty minutes and the longest lasting approximately one hour and a half. This range in interview times is due to their semi-structured nature, wherein a standard guide for interview questions was used but participants were also invited to discuss ideas that were not otherwise asked that may provide meaningful insight to a broader understanding of the topic at hand [29]. The interview guide contained questions organized into four sections: basic background information, including demographics, general health, and past travel experiences; participants’ general decision-making behaviours; participants’ experiences as a medical tourist; and participant feedback on the informational tool.

All interviews were conducted over the phone, a technique that enabled interviews to be completed with Canadians from across the country. While phone interviews are unable to capture participants’ body language, they are increasingly common due to their cost effectiveness [30].

### Data analysis

All interviews were digitally recorded and transcribed verbatim. Transcripts of interviews conducted with participants who had gone abroad for CCSVI treatment were hand coded for four broad categories identified through transcript review by the authors: 1) why CCSVI treatment was sought and where it was obtained; 2) the role of having hope for a cure in seeking CCSVI; 3) the impact of MS on everyday life; and 4) the role other people played in the decision to go abroad. Coding extracts were next independently reviewed by all authors. Following review of these extracts, all authors met to discuss emerging analytic themes, paying particular attention to patterns and outliers in the discussions. During this meeting, consensus was reached on three themes central to participants' decisions to go abroad and the scope of each of these themes was established. In this paper we present a thematic analysis that examines the breadth and depth of these themes. Thematic analysis necessitates identifying themes based on patterns in a qualitative dataset and comparing these emerging themes to established findings in existing literature to help identify their importance or novelty [29].

Following identification of these themes, the first and fourth authors reviewed the full transcripts again to ensure that all relevant data had been captured. The first author then selected the quotes that best illustrated the nature of these themes. All authors independently reviewed these selected quotes and met again to confirm their interpretation along with the scope of each theme and also to discuss the findings as they relate to the existing literatures on medical tourism, patient decision-making, and health care rationing. This study adheres to the RATS guidelines on qualitative research.

## Results

Three themes commonly characterized the journeys of Canadians with MS who decided to leave the country in search of CCSVI therapy abroad. These individuals first experienced a *loss of faith* in the Canadian health system by virtue of their frustration with domestic health care providers and health system administrators who would not accommodate their desires to access unproven interventions at home. Following this loss of faith, these individuals *nurtured hope* that access to CCSVI treatment abroad could eliminate or lessen some of their symptoms of MS, though they were generally measured in their expectations. Once hope for better quality of life through travel abroad was established they finally embarked on their trips abroad, *forming trust* and through it confidence in the credentials and personalized service of their caregivers. Faith, hope, and trust were thus central to their decisions to ultimately go abroad and seek CCSVI treatment in another country as medical tourists. In the sub-sections that follow we examine these three thematic findings in depth.

### Losing faith

The participants described a loss of faith with different aspects of the Canadian health system due to their inability to access CCSVI treatment at home. They felt their diagnosis with MS marked them out as different from the rest of the population and subject to inferior treatment. One participant referred to having the “*scarlet letters of MS attached to my medical file*.” Whereas the participants felt that other Canadians were able to access vascular treatments similar to those used for CCSVI for conditions not related to MS, their doctors told them that they could not apply these same interventions to treat their MS. As a result, one participant “*didn’t have any hope*” after his doctors explained that they “*could lose their job if they even helped me because I have MS*.” In this way, people with MS felt singled out in the public health care system: “*you know they do this procedure on everyone else in Canada but they won’t treat MS patients. Anybody that’s previously labeled, we can’t have it*.” This experience left one participant with the attitude that, “*once you’re labeled with MS, good luck [with accessing care]*.” As a result, the participants felt that their treatment by the Canadian health care system was unfair when compared to the situation of other, similarly situated persons with vascular diseases not related to MS.

Blame for feelings of unfair treatment by the Canadian medical system was largely targeted at neurologists who serve as gatekeepers to specialized treatment. As a result of the perceived resistance of neurologists to the vascular-based CCSVI treatment, one participant voiced that he would not trust a neurologist “*as far as I could throw one*” and another mentioned “*rage and a lot of anger*” targeted at neurologists. This antagonism toward neurological specialists complicated care within the Canadian system for the participants. For example, one participant mentioned not visiting his neurologist before seeking care abroad because all “*neurologists were against CCSVI*.” This disengagement took place following CCSVI treatment abroad as well. One patient said that upon returning to Canada following treatment abroad, his neurologist “*hasn’t talked to me, he hasn’t tried to contact me and I don’t see any reason why I should go see the man*.” Some participants reported tension between the views of their family doctors and neurologists over the potential benefits of CCSVI treatment that created frustration. As a result, one participant described her experience of feeling stymied by her neurologist even when her family doctor was supportive of her accessing CCSVI: “*[the family doctor’s] hands are tied. Because once again a neurologist has domain over my disease. Not even I have domain over my disease…it’s in the hands of the neurologists and that’s not fair*.” Participants were particularly frustrated by their loss of faith in the Canadian medical establishment as they saw CCSVI treatment as a chance to address the root causes of MS rather than continue a cycle of symptom management.

Connected to this loss of faith in Canadian medical specialists, many participants felt a loss of faith in the Canadian health system as a whole and felt pushed into the health systems of other countries as medical tourists. This loss of faith took the form of a violation of the expectation that the Canadian health system would be responsive to their perceived health needs given a lifetime of supporting this system through their taxes and viewing the health system as an important part of their Canadian identity. For some, this experience led to feelings of abandonment, with one participant saying that: “*when you’ve got MS you’re on your own*.” Another noted that he felt as though “*my country abandoned me*,” saying that “*there were actual signs on doctor’s offices that said if you are here to talk about CCSVI please leave*.” In one case, the lack of access to CCSVI at home led a participant to develop an adversarial reaction toward Canadians generally: “*your fellow Canadians would not let you have it at home. So you’re going elsewhere, that’s about all you need to know about your fellow Canadians. …at that point they are adversaries, they’re not working with you, they’re standing in your way*.” In another case, the experience of having to become a medical tourist was described as “*a slap in the face*” by a Canadian health system in which she had had faith throughout her adult life. The financial costs of going abroad for care were another source of anger toward the Canadian health care system that would have paid for the intervention were it offered domestically in the public system: “*I was very upset and disappointed that I had to basically spend $10,000…It just doesn’t make any sense and it’s not fair*.” From the perspective of these participants, the Canadian health system should have provided access to an intervention that they hoped could address the root causes of MS at no additional cost to them. The refusal of specialists, health system administrators, and government officials to provide access to this intervention forced them to go abroad for care at significant personal cost, resulting in an overall loss of faith in the Canadian health system in addition to specific health care providers.

### Nurturing hope

The participants regularly described their decision to travel abroad for an unproven medical intervention as driven by a range of emotions, but particularly as a result of hope for relief of the progressive decline in their health brought on by MS. As one participant described it, CCSVI was “*our only hope at that time*” and the care offered by local doctors did not provide “*a lot of inspiration to carry on*.” Hope for better health from CCSVI treatment was also discussed in terms of providing “*positivity*” and for bringing about a sense of excitement. For example, a participant described how hope combined with excitement shaped her interactions with the doctor she saw while abroad. This doctor “*didn’t make any, any real promises he just had as much hope as I did and that was, it was nice, it was exciting, more than I had had in a long time*.” While hope drove many to seek CCSVI treatment abroad, contrasting emotions such as fear were experienced as well, with one participant explaining that there was also “*the fear of the unknown*.” Despite some fear at the prospect of traveling abroad to access an unproven medical intervention, their new hope of arresting the progression of their MS was the dominant emotion driving participants’ decisions to become medical tourists.

Worsening health and the loss of quality of life brought on by MS was a significant factor driving participants abroad. This loss of quality of life was the key motivation for some who reported being “*willing to do anything to have the quality of life that you want*.” Given the progressive loss of quality of life and the perceived lack of effective treatments within Canada, seeking CCSVI treatment abroad was felt by some to be the only option for treatment, even if success was far from guaranteed. Despite this lack of guaranteed success, doing nothing was not seen as an option. For these participants, staying in Canada would be equivalent to accepting as permanent the loss of function from MS, and so any response was desirable. As one participant put it: “*I knew what was going to happen to me if I did nothing. And so I was going to do something*.” For others, not seeking treatment abroad would be the same as accepting death, thus making taking a chance on CCSVI treatment “*a rational choice*.” Due to the progressive nature of some forms of MS, many participants felt pressure to act quickly rather than wait for the possibility that CCSVI treatment would eventually be offered domestically. As one noted, *“time works against you when you have MS, every day there is a little piece of you that seems to die off.*” The natural progress of their disease and restrictions on access to CCSVI treatment at home, then, made the decision to go abroad for care seem clear and fully rational.

For the most part, the participants did not believe that they would be cured of MS as a result of undergoing CCSVI treatment abroad. Their measured hope was in some cases informed by the cautions of the physician providing the intervention. In one case, an international physician told a patient: ‘*don’t go in expecting miracles. This is not a cure, this is symptom management*.’ In another case, a patient did not receive the symptom relief that she most desired, but still saw an improvement in other areas. As a result, she reported that her “*overall well-being has improved*.” For another participant, symptom relief did not need to be permanent in order for the procedure to be deemed worthwhile: “*if there was a chance that a person could alleviate the symptoms for even a little period of time. It’s worth it*.” Some participants had their expectations around CCSVI treatment tempered by past experiences with unproven interventions, having previously been told of “*quack remedies*” or actually spending money on “*trying for hope*.”

### Forming trust

While loss of faith in the Canadian health system and hope for improved health both motivated participants to travel abroad for care, they had to develop some form of trust in the specific clinics and physicians in the destination countries they visited. For some, the decision to go abroad for care was supported in part by peer support networks that participants had built up with others seeking, or who had sought already, CCSVI treatment abroad. Distrust of neurologists and the MS Society led one participant to focus on the recommendations of fellow people with MS because it “*is our disease*” and they are “*banding together*.” Within online groups, the fact that people who had already gone abroad had “*firsthand knowledge*” of the intervention was seen as important. Online groups on Facebook became (and continue to be) trusted sources of information for some participants, with one noting that “*we were able to go to this [Facebook] group and find all of these good places and bad places to go to get it done*” and another likening Facebook to the “*underground railroad*.” Many participants found confidence in their own research, trusting themselves to determine which sources of information were most reliable. One noted how seriously she had researched CCSVI treatment and the clinics abroad, saying: “*I don’t just go off on a song and a prayer, I actually research things and then I make a decision*.” In all of these cases, the participants we spoke with became heavily reliant on their own networks, both face-to-face and virtual, and online information when determining where to travel for care and which physicians and clinics to place their trust in.

While most participants had lost faith in the Canadian medical system and especially the neurologists treating them, the professionalism and credentials of their physicians abroad remained an important element in building trust in the care they would receive. This process of trust building took place in a context where several participants discussed having read media reports of Canadians suffering significant negative health impacts from accessing CCSVI treatment abroad. In one case, a participant noted that her physician abroad was “*actively researching*” new MS treatments and saved the life of another patient while she was in the facility. Another took pictures of the plaques and credentials on the wall of the waiting room in the facility, noting “*a huge wall full of certificates*.” Many participants felt able to assess the credentials of their physicians abroad prior to travel, often with assistance from online information and their support networks. Phrases used to assess these credentials included “*board certified*” and “*experts*,” with one participant noting that she “*trusted the qualifications that they said these people had*.” The opinions of physicians back home also served to instill confidence in physicians abroad. In one case, a participant asked her regular family physician about the credentials of her prospective physicians in India and was told that they would have been educated in the United Kingdom and so their training would be similar to that of Canadian doctors. The ability to communicate with physicians abroad prior to booking CCSVI treatment was also important to building confidence in the quality of care they would receive, and trust in these physicians. After she spoke with her international physician over the phone, one participant noted that: “*I felt very, very confident*.” However, for a minority of these participants, trust in professionalism was more of a leap of faith based on “*faith and trust*” that the physician was looking out for their best interests rather than being based on degrees and other qualifications.

The interviews revealed that participants not only needed to trust the physicians from whom they received CCSVI treatment, but also that they needed to form a certain degree of trust that their health and safety were secure in the destination country and clinic as well. In some cases, participants were already familiar with the international health systems being accessed, with one choosing a destination based on having been born there and having other family connections to that country. Another participant felt comfortable traveling to Poland as that country had joined the European Union and adopted Western medical standards, which he trusted as a quality indicator. Some participants were initially concerned about the quality of care and facilities abroad. One participant traveling to Bulgaria was reassured that the hospital had been built and funded by Japan. Another participant had heard “*weird things about Mexico*” and was worried that she would find “*a dirty stable*” instead of a hospital. She was ultimately reassured by the appearance of cleanliness of the facility and that “*nurses down there, they’re dressed like nurses with white caps on and starch white gowns*.” In other cases, participants felt that the standard of care they received abroad was superior to that experienced in Canada, which enhanced their trust in clinics abroad. One patient noted her physician abroad “*spent probably forty-five minutes to an hour on the phone with me. I don’t even get forty-five minutes to an hour with my doctor here in Canada*.”

## Discussion

The findings shared above show that a number of complex factors surrounding losing faith, nurturing hope, and forming trust informed participants’ decisions to go abroad for CCSVI treatment, including the country they visited, the clinic they sought care in, and the physician who provided this care. Participants felt abandoned by the Canadian health care system as well as their neurologists, which prompted them to lose faith in both the system as well as specific providers. In light of this loss of faith and driven by the desire for symptom relief, they felt as though they had no other option but to go abroad for treatment. Overall, hope for an improved quality of life drove participants’ decisions to travel abroad for the unproven CCSVI procedure. This was not hope disconnected from reason, however, and many were modest in their expectations and cognizant that improved quality of life was not guaranteed. For some, simply trying to better manage a particular symptom was enough of a motivation to go abroad. Despite their loss of faith in medical professionals at home, the credentials and opinions of medical experts still mattered greatly to their willingness to trust their health and safety to CCSVI treatment. Whereas they had lost faith in Canadian specialists, they were willing to place trust in foreign medical providers, often aided by the experiences and perspectives of their peers and the familiarity of the facilities abroad. These findings raise a number of important issues around the shifting of blame and enactment of personal responsibility for care as well as the balance between hope and rational choice that we examine in this section.

### Shifting blame and personal responsibility

Characteristic of these interviews were feelings of neglect and abandonment by the Canadian health system, specialists within that system, and even the Canadian public. These participants felt extreme frustration that a procedure that was being offered in other countries was not being offered in Canada. Similar frustration has been reported by Canadians who have gone abroad as medical tourists for orthopaedic procedures that are not readily available at home [31]. In the current study, this frustration was fueled by the fact that refusal to offer CCSVI treatment was not a result of a lack of expertise but because of an active choice by political representatives to block access to this intervention. This frustration was exhibited despite the lack of proven efficacy of CCSVI treatment in addressing the causes of MS and the fact that the government of Canada was not alone in not funding access to and warning against choosing to undergo CCSVI treatment [9–14]. For many, this decision by the government to block access to CCSVI treatment was experienced as a loss of agency – where agency is the state of being that enables us to have the ability and flexibility to problem solve [32]. This reality thrust the participants into becoming extremely active proponents of their own health, seeking out new interventions for MS on their own, communicating with similar others in person and through online communities, and sometimes even advocating for increased funding for research and access to care. A number of studies focused on the lived experience of chronic illness have documented similar individual and collective activities being undertaken by those managing chronic illnesses such as MS [33, 34]. It is not surprising, then, that many of these participants were unwilling to abandon their active engagement with managing their disease, choosing instead to seek access to CCSVI treatment abroad even if it required significant personal risk, inconvenience, and financial cost.

Diagnosis with MS is an extremely emotionally charged event [35]. While many of the participants we spoke with experienced feelings of anger and resentment as a result of their diagnosis, they remained active in seeking, if not a cure for their disease, at least access to the latest interventions that could enhance the quality of their lives. As a result of their inability to access CCSVI treatment within Canada, some of these feelings of anger and resentment shifted from their disease to the Canadian health system. Other research has shown that the development of such resentment leads Canadians to view their engagement in medical tourism as ethically defensible [36]. In many cases participants sought specific targets for their frustration, including neurologists who sought to keep MS defined as a neurological rather than vascular condition and a Canadian public who simply did not care about their needs. As a result, many of these participants exhibited an ‘us vs. them’ attitude, alienating them from their domestic health care system. Similar findings have been reported regarding the health care experiences of individuals diagnosed with contested chronic illnesses such as fibromyalgia syndrome or myalgic encephalomyelitis, wherein such individuals have been made to feel at odds with the Canadian health system [37, 38]. In the current study, this feeling of alienation from the health system was heightened by participants’ belief, gained from active research about their disease and CCSVI treatment, and bolstered by an active online community of fellow sufferers, that a treatment for their disease existed, if only their government and health system administrators would stop actively blocking them from accessing it at home.

As a result of their decision to seek care abroad, participants took control of their own health from a system that uses gatekeepers to control access to care and placed responsibility for their health into their own hands. In many senses, this shift was empowering, allowing participants to choose not only what procedures they would be given, but by what doctor, in what facility and country, and at what time. In a similar manner, the global medical tourism industry has been framed by some as enabling patient empowerment as it facilitates choice and access, though at a cost [39]. Online communities of people with MS aided in this process of empowerment by exchanging tips about the best physicians, which facilities had the shortest wait times for care, and which countries were becoming new destinations for Canadians seeking CCSVI treatment. The downside of this personal empowerment, however, was a shift in responsibility for any negative health consequences to the participants themselves as a result of seeking care abroad privately, and often without the support of their regular physicians at home. The Canadian media contains many stories of people who have obtained CCSVI treatment abroad returning to Canada having negative health consequences and being denied care by domestic physicians [25, 40, 41]. Many of the participants we interviewed specifically cited these stories and were aware that by going abroad for care they were potentially endangering their future access to follow-up or emergency care at home. More importantly, they saw their decision to go abroad for care as creating a permanent rift with the Canadian health system. That they were willing to take on this responsibility speaks to their drive to improve their quality of life, frustration with the Canadian system, and desperation for gaining access to new care options.

### Hope and rational choice

The findings illustrate the distinction between participants’ hope and expectations when undergoing CCSVI treatment. Here hope can be understood as an individual’s assessment of the most *desirable* outcomes while expectations can be understood as an individual’s probability-driven assessment of the most *likely* outcomes from medical treatment. Researchers have highlighted the therapeutic importance for health care providers to balance fostering hope and providing realistic expectations to patients [42]. Finding this balance can be challenging as these concepts are often conflated. People may understand that the desirable outcomes differ from the probable outcomes, yet hope persists as a coping mechanism. In the current study, the participants’ understanding of MS as being incurable and degenerative appeared to inform the development of hope as a coping mechanism, which played a significant role in leading them to consider going abroad for CCSVI treatment. Hope is also both shaped by and shapes one’s agency [32]. Participants’ discussions of trip planning demonstrate an example of this mutually reinforcing process as the agency they exerted in traveling abroad for CCSVI therapy fostered hope for symptom-relief, while hope for symptom-relief fostered increased agency to pursue goals that were not merely determined by the expected health outcomes.

Contrary to common assumptions that individuals engage in unproven procedures due to a lack of awareness of likely procedure outcomes or potential risks [42], the findings shared here show that people traveling abroad for unproven procedures can embark on a journey that places these risks within a context of hope and even possibility. As has been shown in research focused on people traveling abroad for unproven stem cell therapies, this journey is thus undertaken within the context of considerations of both the risks and possibilities that shape individual hope that ultimately inform decisions in medical tourism [43]. The findings of the current study demonstrate the ability for this journey to bring about excitement and feelings of empowerment that shape individual’s considerations of risks and possible outcomes. Although there is concern that a strong sense of hope may push people to overlook health and safety risks when making health decisions [44], there is also recognition that the empowerment, optimism, and motivation fostered through hope can provide important therapeutic benefits for people coping with chronic illness [32, 43]. Although the current study did not assess the therapeutic benefits of the hope expressed by the participants, the findings do point to a need to better understand the role and benefits of hope in motivating medical tourists’ journeys abroad in order to complement the existing significant research focused on risk (e.g., [45–47]).

The findings demonstrate that participants’ hope was tempered with rational expectations about their CCSVI treatment outcomes. Expectations of major improvements in health or wellbeing were often tempered by physician advice, the experiences of others, or negative past experiences. Instead, improvements in fatigue, bladder control, or short-term changes in general wellbeing were expectations deemed sufficient enough to justify their choice of intervention. Whether these outcomes would have been identified as indicators of success prior to receiving the intervention, or are a reflection of individuals’ desire to attribute success to CCSVI treatment in the presence of negligible changes in health or wellbeing, is a research question beyond the scope of our study. However, given that all participants perceived their CCSVI treatments to be successful, something that further reinforces feelings of neglect by the Canadian system, differences between individual and societal determinations of ‘value for money’ in health care are important considerations to be made in relation to providing access to unproven care.

While the therapeutic benefits of hope and experiences of individuals like those who participated in this study should play a role in decision-making around the distribution of health resources in a public health system such as Canada’s, it is important to stress that supporting the hope and empowerment of individuals cannot alone justify utilizing limited health resources for providing access to an unproven medical intervention such as CCSVI treatment. Putting resources into CCSVI treatment would likely necessitate a loss of funding for other interventions where evidence backs their efficacy in improving health. CCSVI treatment has not been found to be effective in treating MS nor to have a clear biological basis for doing so. [8–14]. Those accessing CCSVI abroad have encountered health problems as a result of this intervention, meaning that the net effect of access to CCSVI could be negative for many Canadians with MS [25, 26]. Additional research is needed to better understand the impact of CCSVI treatment on MS and other chronic diseases, but this research is not supported by individual patients receiving these interventions outside of clinical trials [8]. Several jurisdictions in Canada have provided access to these trials, granting some relief to Canadians with MS who wish to access CCSVI treatment (through certainly less than enough to meet demand for this intervention) [20]. Thus, despite the experiences of the participants in this study and benefits they may have received as individuals by accessing CCSVI treatment abroad, the decision within Canada not to provide access to CCSVI treatment outside of clinical trials is very well supported on grounds of patient safety and the efficient distribution of health resources.

### Limitations

This study helps to give voice to the decision-making and experiences of Canadians seeking an unproven medical intervention outside of their home country. The insights described here provide insights for policy makers seeking to address the loss of hope felt by these patients without abandoning the principle that scarce public resources should not be used to fund unproven interventions. While these findings are relevant for other jurisdictions and other unproven interventions, the experiences of these patients are highly informed by the public nature of the Canadian health system and the gatekeeper role played by family physicians and neurological specialists in that system, elements not shared by other health systems. Moreover, as the study from which this paper is derived did not set out to explore the decision-making of this particular patient group, dimensions of their experience may not be represented here. Additional study of the experiences of individuals seeking unproven interventions abroad is warranted.

## Conclusions

The globalization of trade in health services will continue to provide opportunities to access unproven medical interventions not available in individuals’ home countries. Canadians seeking CCSVI treatment is a high profile example of this practice, but they are only one of many groups worldwide traveling abroad for unproven interventions. Given the dangers associated with some of these interventions, questions about their efficacy, and difficulties accessing follow up care, it is easy to treat those paying out of pocket for these interventions as vulnerable and even gullible individuals that should be protected from themselves. However, the perspectives of the participants discussed here demonstrate that the story can be much more complex. These participants had lost faith in their home health care system but placed trust in other health professionals in order to nurture hope for improved wellbeing. They were for the most part measured in their expectations and felt renewed hope by seeking medical care abroad.

These findings are important for health professionals and policy makers in two respects. First, the loss of faith of these participants should raise questions about what more can be done to restore this faith in both Canadians with MS and other groups globally. Second, while action must be taken to protect individuals from harm from potentially dangerous and unproven medical interventions, consideration should also be given to the benefits, including restored hope, for some individuals accessing these interventions. While accommodating the desire to access unproven and potentially dangerous interventions using public funds is inappropriate, this study demonstrates the need to better address the alienation felt by these patients. The context of Canadians accessing CCSVI treatment is unique, but these lessons are also relevant for other groups accessing unproven interventions abroad. Continued study of the perspectives of these groups will help health professionals and policy makers to craft responses to these practices that are more responsive to the experiences of these individuals.
